# Trust, risk and organic food: Evidence from the UK and Japan

**DOI:** 10.1007/s13165-026-00547-7

**Published:** 2026-02-28

**Authors:** Steven David Pickering, Martin Ejnar Hansen, Yosuke Sunahara

**Affiliations:** 1 University of Amsterdam, Postbus 15578, 1001 NB Amsterdam, Netherlands; 2 Brunel University of London, Kingston Lane, Uxbridge, UB8 3PH UK; 3 Kobe University, 2-1 Rokkodai-Cho, Nada-Ku, Kobe, 657-8501 Japan

**Keywords:** Organic food, Willingness to pay, Trust in government, Risk appetite, Japan, United Kingdom

## Abstract

Why do some consumers choose pricier organic options while others pass them by? Drawing on large, harmonised survey samples from the United Kingdom (*n* = 1,276) and Japan (*n* = 1,532), we examine whether three psychological factors (generalised social trust, trust in government and individual risk appetite) are associated with willingness to pay more for organic dairy, meat, eggs and vegetables. Logistic regression models show that trust in government is the most consistent correlate of willingness to pay more for organic food, with a stronger association in Japan than in the UK. Generalised social trust is more closely linked to perceptions that organic production aligns with personal values, while individuals with higher risk appetite are consistently more open to paying a premium, regardless of country or food type. Taken together, trust and risk orientations explain meaningful variation in organic preferences after controlling for age, education, gender and ideology. These findings suggest that efforts to expand organic food markets may benefit from pairing credible certification systems with communication strategies that reduce perceived risk, particularly in institutional contexts characterised by high levels of public trust.

## Introduction

Global concerns over food safety, health and sustainability have pushed organic products from niche to mainstream. Yet consumers cannot inspect farming practices directly; organic food is therefore a classic credence good, requiring consumers to rely on labels, institutions and social cues. Scholars have charted many drivers of organic uptake, such as environmental concern, health motives and demographics (Vega-Zamora et al. 2020; Yang et al. 2023). Although recent work has examined trust and risk in consumer behaviour (particularly in relation to noumenic or trust-mediated food attributes: see Boccia and Punzo 2020; Wu et al. 2021; Yu et al. 2022; Hu et al. 2024; Meira et al. 2024), most studies focus on product attributes, advertising effects, or perceived quality rather than on broader social or institutional dimensions of trust. Research that directly links generalised and institutional trust, together with individual risk orientation to organic food preferences therefore remains limited (though see Esfandiar et al 2025; Macready et al 2025).

Recent work has begun to address the psychological underpinnings of organic food choices, for example by examining the attitude-intention gap through behavioural reasoning theory and subjective norms in the Vietnamese context (Nguyen and Nguyen 2025). Building on this literature, our study brings a cross-national perspective to the intersection of trust, risk, and organic consumption, allowing us to assess whether relationships identified in single-country studies generalise across different institutional and certification contexts.

The issue is especially salient in two contrasting markets: the mature, multi-certifier United Kingdom and the smaller, state-certified Japanese sector. Drawing on large, nationally quota-balanced surveys (UK = 1,276; Japan = 1,532), we examine how different forms of trust and individual risk appetite influence consumers’ willingness to pay for organic food, perceived product quality, and value alignment across these contexts.

Consumers cannot directly observe the production of organic goods; they must rely on information intermediaries such as labels, institutions, and social cues. Scientific experts and regulatory bodies play a central role in shaping public opinion towards organic food (Sønderskov and Daugbjerg 2011; Ladwein and Romero 2021; Shahabi Ahangarkolae and Gorton, 2021). The growing concerns about environmental sustainability have also increased the awareness of the public towards organic food and increased the focus on certification processes of organic food (Murphy et al 2022; Park-Poaps and Han 2025). Trust plays a significant role in this relationship, encompassing both generalised interpersonal trust and more specific forms of institutional and policy-related trust (Ayyub et al. 2021; Wu et al. 2021; Morosan et al. 2025; Macready et al. 2025). These trust orientations do not exist in a political vacuum. Political attitudes have increasingly shaped domains not traditionally viewed as political, including food consumption. Individuals with more left-leaning ideological standpoints are generally more likely to favour organic food, while right-leaning individuals tend to express greater scepticism towards environmentally friendly and organic products (Saraiva et al. 2021; Tiganis et al. 2023).

The expansion of organic food consumption has not occurred evenly across the globe (Seegebarth et al. 2016). The United Kingdom and Japan offer contrasting contexts in this regard (Ashkenazi and Jacob 2003; Mason 2004). The UK has a mature organic food market supported by both public and private certification bodies. In contrast, Japan's organic sector remains relatively small, with the Japan Agricultural Standards (JAS) scheme serving as the primary source of certification. These institutional differences provide a useful comparative setting for examining how trust in certification and institutions, together with individual risk perceptions, shape organic consumption under different regulatory regimes.

Food neophobia, the fear of trying new or unfamiliar foods, also influences consumer behaviour toward organic products (Siddiqui et al 2022; Park-Poaps and Han 2025). This hesitation reflects a form of risk aversion linked to trust in certification and safety standards. Such aversion is closely tied to levels of trust, making trust a pivotal factor in mitigating perceived uncertainty surrounding organic consumption. Furthermore, risk-taking behaviour itself is often conditioned by trust in institutions and certification processes (Cruwys et al. 2021; Siegrist & Cvetkovich 2000). Individuals with a greater willingness to take risks may therefore be more inclined to view organic labels and certifications as credible assurances of quality and safety.

Trust and risk underpin our core research focus. Specifically, we ask:

1. How are trust levels and risk appetite associated with consumer preferences for organic food in the United Kingdom and Japan?
2. Do these relationships extend beyond willingness to pay, shaping perceptions of product quality and alignment with personal values?
3. To what extent are trust and risk associated with organic food preference across the two countries?

We focus on three central outcomes: (1) consumers’ willingness to pay a premium for organic food, (2) their belief in the consistency and quality of organic products, and (3) the extent to which organic food reflects personal or societal values. Our three main explanatory variables are generalised social trust, trust in government, and risk appetite, with controls for age, sex, education, and political ideology. By combining insights from political trust research with consumer behaviour studies, the article contributes a comparative framework for understanding how institutional confidence and risk orientation jointly shape sustainable consumption.

## Literature review

Generalised trust refers to the belief that “most people can be trusted.” In food consumption, this form of trust can enhance consumers’ confidence in product claims, even in the absence of institutional cues. Sønderskov (2009) found that generalised trust facilitates cooperative behaviour, including sustainable consumption, by reducing the perceived risk of free-riding or misinformation. Vega-Zamora et al. (2020) further argue that value-driven consumers who trust others are more likely to purchase organic food as a form of ethical consumption, a pattern consistent with earlier work showing that attitudes, perceived benefits, and trust shape acceptance of value-laden foods (Verbeke 2005). Recent work also points to the particular notion of “green trust” (Rashid and Lone 2024). While generalised trust can enhance confidence in producers and peers, its effects may be moderated by institutional strength. Seegebarth et al. (2016) show that cultural trust norms shape perceptions of organic value across national segments. Macready et al. (2025) argue that trust is a central moderating factor between motivation and intention for purchasing sustainable foods. In societies with strong informal norms and high generalised trust, consumers may rely more on social proof and word-of-mouth; in lower-trust contexts, formal certification may play a stronger role. Trust is a key concept in how individuals interact with each other and with institutions. From a conceptual perspective, trust is not unconditional and can be divided into distinct forms, most notably generalized and specific trust (Freitag and Bühlmann 2009; Freitag and Traunmüller 2009). While generalized trust is associated with broadly desirable outcomes, specific trust is situational, experience-based and sensitive to external events and institutional performance (Levi and Stoker 2000).

It is important to remember that organic food relies on attributes that are not directly verifiable by consumers (Schleenbecker and Hamm 2013). This lack of direct observability makes trust in the food system, including producers, certifiers and regulators, key to consumer decision-making (Murphy et al. 2022; Ladwein & Romero 2021). Trust enhances perceived credibility of labels and reduces uncertainty, thereby influencing both intention and actual behaviour (Ayyub et al. 2021; Ronteltap et al. 2007). Information also plays a central role, with trust shaping how consumers interpret and evaluate information about organic products (Yuan et al. 2024). A large body of research highlights the role of consumer trust in certification systems as a key driver of organic food choice. For instance, Murphy et al. (2022) found that trust in organic certification significantly predicted positive consumer attitudes across four European countries. Similarly, Ayyub et al. (2021) showed that trust mediates the relationship between health consciousness and organic food purchase intention in a developing context, similar findings reported by Arslan (2025). As Sønderskov and Daugbjerg (2011) note, consumer confidence in eco-labelling schemes tends to be higher when the state plays a prominent role in monitoring and enforcement. Ladwein and Romero (2021) found that trust in institutions, in particular regulators, mediates consumer relationships with producers and retailers of organic food. Recent research has also highlighted the need to account for psychological and social factors beyond trust. For example, Nguyen and Nguyen (2025) develop an integrated framework that combines behavioural reasoning theory with injunctive and descriptive norms to explain organic purchase intentions in Vietnam. Their findings underscore the importance of social influence and context-specific reasoning alongside trust in understanding organic food uptake.

Trust in government is therefore an important factor in understanding consumer preferences for organic food. In Japan, institutional trust is central to food-related decision-making, though perceptions of government effectiveness in organic certification vary (Yang et al. 2022; Tandon et al. 2021). Hu et al. (2024) show that consumers who trust regulatory bodies are significantly more willing to pay a premium for organic food. Similar findings are reported in the South Asian context (Yang et al. 2023), where institutional trust supports both intentions and consumption. In the UK, organic certification is pluralistic, with both governmental and third-party actors like the Soil Association involved. This can lead to complex trust dynamics: consumers may rely on brand recognition, peer influence, or generalised trust in addition to institutional assurance (Kowalska et al. 2021). Japan presents a different case. Trust in public institutions remains high, but organic food is less mainstream. Yang et al. (2022) found that Japanese consumers’ trust in government significantly predicted willingness to pay for environmentally friendly products. However, due to the relative unfamiliarity of organic food, many Japanese consumers view organic claims with scepticism unless reinforced by strong institutional endorsement (Tandon et al. 2021).

Risk perception plays a pivotal role in shaping consumer behaviour towards organic food, given its properties as credence goods (Giannakas 2002; Finucane & Holup 2005). As a result, trust and perceived risk are closely intertwined factors influencing organic food choices. Pandey et al. (2020) demonstrate that risk perception varies significantly across income groups. Low-income consumers are more risk-averse due to the higher price premium associated with organic food and often require assurances such as money-back guarantees or liberal return policies. In contrast, high-income consumers are less sensitive to financial risk and instead rely on product and service quality to develop trust in retailers, using trust as a proxy to mitigate risk (Pandey et al. 2020).

Beyond economic considerations, risk perception is also shaped by beliefs, trust in science and cognitive processes. Meira et al. (2024) examine risk perception in relation to consumer purchase intention for produce produced with pesticides. Koswatta et al. (2023) expand on the complexity of public perception by illustrating how beliefs, trust in science and cognitive dissonance influence acceptance of information about organic food. Despite scientific ambiguity over the actual health benefits of organic food, many consumers perceive it as healthier and safer (see also Macready et al 2025). This discrepancy fosters risk aversion in those who distrust scientific institutions or experience dissonance when new evidence conflicts with deeply held beliefs, potentially dampening willingness to purchase (Koswatta et al. 2023). Perlstein (2024) broadens this understanding by emphasising the socially constructed nature of risk. Interpersonal discussions, both offline and online, amplify or attenuate individual risk perceptions. These interactions often reinforce existing attitudes, contributing to shared norms that either increase aversion to risk or enhance willingness to buy. Thus, social context significantly modulates risk appetite (Perlstein 2024). Hilverda and Kuttschreuter (2018) examine how perceived risks around organic food are shared online, as also suggested by Park-Poaps and Han (2025). Although the actual intention to share such information is low, injunctive norms and outcome expectancies drive online risk discussions. Their findings indicate that higher risk perception correlates, albeit weakly, with a greater likelihood of information sharing, which may influence wider consumer attitudes (Hilverda and Kuttschreuter 2018).

Taken together, this literature aligns with a growing body of work conceptualising organic and sustainable foods as credence goods whose consumption decisions are shaped by trust, perceived risk and institutional context rather than by directly observable attributes (Boccia et al. 2018; Boccia & Sarno 2019; Boccia & Punzo 2020). Much of this research has relied on structural equation modelling or discrete choice experiments to examine how trust, values, and risk perceptions interact to shape willingness to pay and purchase intentions. While these approaches offer important insights into latent mechanisms, our study adopts a complementary strategy by focusing on observed associations between trust, risk appetite, and multiple outcome dimensions across two contrasting regulatory environments. In doing so, we extend this literature by examining whether relationships identified in single-country or product-focused studies generalise across institutional contexts characterised by different certification regimes.

Accordingly, we propose three sets of hypotheses, based on generalised social trust, trust in government and risk appetite. Drawing on the literature reviewed above, these hypotheses examine whether trust and risk orientations are consistently associated with organic food preferences across different outcome dimensions and institutional contexts. While the United Kingdom and Japan differ in their regulatory and market structures, we test whether the direction of these relationships is similar across both cases.

### Willingness-to-pay hypotheses

- H1a: Higher generalised social trust is associated with greater willingness to pay a premium for organic food.
- H1b: Higher trust in government is associated with greater willingness to pay a premium for organic food.
- H1c: Higher risk appetite is associated with greater willingness to pay a premium for organic food.

### Perceived quality hypotheses

- H2a: Higher generalised social trust is associated with stronger belief in the consistent quality of organic food.
- H2b: Higher trust in government is associated with stronger belief in the consistent quality of organic food.
- H2c: Higher risk appetite is associated with stronger belief in the consistent quality of organic food.

### Value alignment hypotheses

- H3a: Higher generalised social trust is associated with a stronger belief that organic food reflects personal or societal values.
- H3b: Higher trust in government is associated with a stronger belief that organic food reflects personal or societal values.
- H3c: Higher risk appetite is associated with a stronger belief that organic food reflects personal or societal values.

## Data and methodology

We use a series of survey questions conducted in April 2024 in Japan (via Rakuten Insight) and April–May 2024 in England (via YouGov). The final samples include 1,532 respondents in Japan and 1,276 in England. Both survey agencies maintain large online panels and applied standard national quotas for age, gender, and region to approximate population distributions. The demographic profiles of both samples broadly reflect national population structures, with only minor imbalances such as a slightly higher proportion of women among UK respondents (see Table 2). As with most online panel surveys, the samples are not probability-based; however, quota controls help ensure reasonable comparability across key demographic dimensions. The surveys are therefore considered suitable for analysing attitudinal relationships rather than estimating precise population parameters.

For the willingness to pay more for organic food, we asked the respondents the following question: “Foods are certified as organic if they meet certain legal standards. Would you be willing or unwilling to pay extra for organic versions of the following foods?”. The respondents were then given five food types for which they had to indicate whether they would pay more or not. The food types were: Dairy products, Beef, Chicken, Eggs, and Vegetables. This approach captures a clear and interpretable threshold of willingness to pay, rather than eliciting precise monetary valuations. Our variable takes the value 1 if the respondent was willing to pay more for organic food for the particular food type and 0 if that is not the case. We present the percentage of respondents in agreement with each food type for each country in Table 1.

**Table 1:** Percentage of respondents willing to pay more for organic foods (percentages are based on those who consume the foods mentioned)

Willing to pay more for organic:	UK (%)	Japan (%)
Dairy products	45.22	39.14
Beef	41.54	36.64
Chicken	46.16	38.19
Eggs	48.59	40.94
Vegetables	46.55	43.43

As the willingness-to-pay outcomes are binary, we analyse them using logistic regression models, which estimate the independent effects of trust, risk, and control variables on the probability of being willing to pay more for organic food. For the continuous attitudinal outcomes (quality and values), we use linear regression models to allow straightforward interpretation of coefficient direction and magnitude. This modelling strategy prioritises transparency and comparability across outcomes and countries, while avoiding additional assumptions about latent constructs or choice structures required by more complex techniques.

While willingness to pay provides insight into behavioural intentions, we also examine whether respondents believe that organic food is consistent in its quality and whether its production aligns with their personal values. These dimensions are measured using the following question: “Thinking about your regular food shop, and your preferences in this regard. To what extent do you agree or disagree with the following statements?” Respondents were presented with two statements: “Organic food is of consistent quality” and “Organic food is produced with values that I believe in”. Responses were recorded on a seven-point scale ranging from 1 (Strongly Disagree) to 7 (Strongly Agree). This mixed outcome strategy allows each dependent variable to be analysed on its appropriate scale while maintaining comparability across the two national contexts. Full replication data and code are available from the Harvard Dataverse at 10.7910/DVN/Q8KOW8.

For all analyses we included three main independent variables. First, we include a variable for measuring the respondent’s level of general social trust using the question: “Generally speaking, would you say that most people can be trusted, or that you can’t be too careful in dealing with people?” This is measured on a seven-point scale ranging from 1 "You cannot be too careful" to 7 "Most people can be trusted". For our measure of trust in government we asked: “Using a scale of 1 to 7 where 1 means "not at all" and 7 means "completely", how much do you trust the Government?” For risk appetite we asked respondents: “Generally speaking, how willing are you to take risks?”, with responses recorded on a scale from 0 (extremely unwilling to take risks) to 10 (extremely willing to take risks). All three measures are treated as continuous variables in the analysis.

We also include four control variables, specifically age (measured in years), whether the respondent completed university education or not, whether the respondent is a woman and how they place themselves on a left–right scale measured from 0–10. The descriptive statistics for our two other dependent variables, our independent variables and our control variables can be seen in Table 2 below.

**Table 2:** Descriptive statistics

Variable	UK	Japan
Trust Government (1 to 7: mean, SD)	2.68 (1.53)	3.10 (1.48)
General trust (1 to 7: mean, SD)	3.57 (1.61)	3.11 (1.55)
Take risks (0 to 10: mean, SD)	4.85 (2.14)	4.64 (1.96)
Left–right (0 to 10: mean, SD)	4.85 (2.16)	4.99 (1.55)
University education	38.9%	50.0%
Women	55.3%	49.8%
Men	44.7%	50.2%
Age (range, mean, SD)	18–88, 49.38 (17.92)	18–79, 50.20 (16.19)
N	1276	1532

## Results

**Fig. 1:**
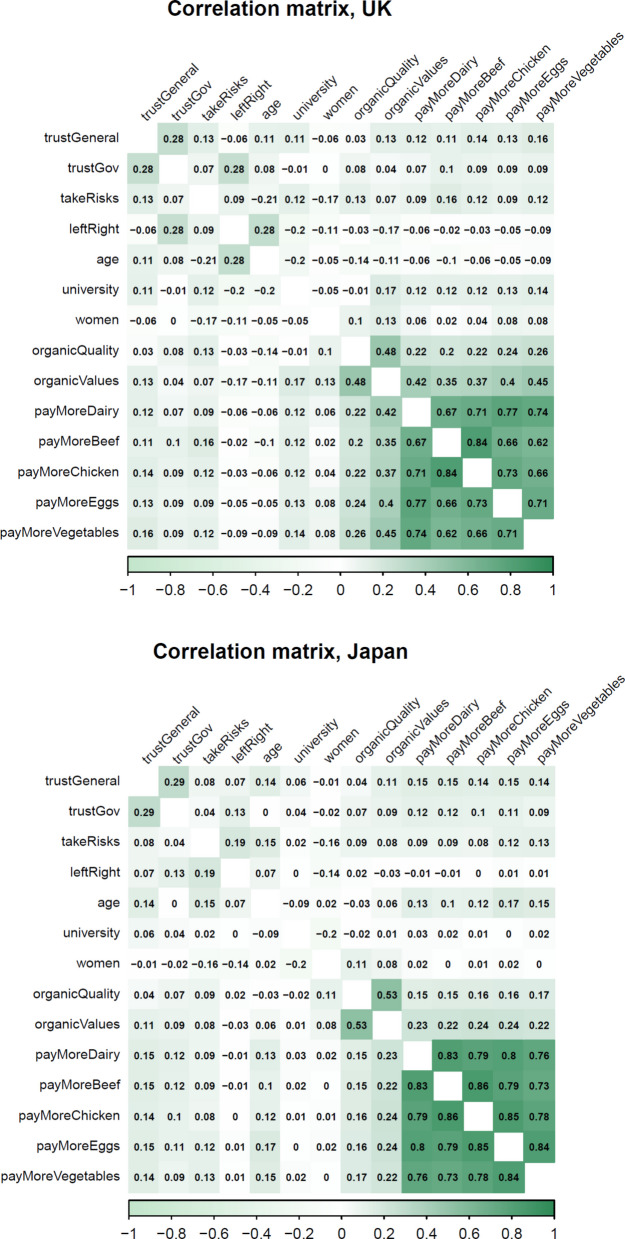
Correlation matrices for all study variables in (**a**) the United Kingdom and (**b**) Japan. Cells display Pearson’s r with pairwise deletion; shading intensity indicates magnitude and direction (green = positive, white = weak). Binary variables (university, women, and the five willingness-to-pay items) are represented by point-biserial correlations

We present our results in two stages. First, because our primary dependent variable (willingness to pay more for organic food) is binary, we begin by examining the patterns of association among all study variables to contextualise the subsequent regression results. Figure 1 displays the bivariate correlation matrices for the United Kingdom and Japan, covering all predictors, control variables and willingness-to-pay items. These visual summaries serve two purposes: they provide a descriptive overview of the relationships between trust, risk appetite and attitudes toward organic food, without implying causal direction, and they allow us to assess potential multicollinearity among the predictors.

The matrices show that generalised and institutional trust are moderately correlated, indicating that they capture related but distinct dimensions of trust. Associations between trust and organic outcomes (perceived quality, value alignment, and the five willingness-to-pay items) are positive but modest. Risk appetite is also weakly and positively related to willingness to pay, particularly in the UK, suggesting that people more comfortable with uncertainty are slightly more likely to pay a premium for organic products. As expected, the five willingness-to-pay variables form a highly correlated block, confirming that they represent a single underlying disposition toward paying more for organic food. The patterns appear broadly similar across the two countries, suggesting that while national contexts differ, the underlying relationships among trust, risk and organic food attitudes are comparable.

Before turning to the regression analyses, we compared mean levels of the key variables across countries (Table 3). Trust in government was significantly higher in Japan than in the United Kingdom, whereas generalised social trust was higher in the United Kingdom. Risk appetite was slightly higher among UK respondents, while ideological self-placement showed only marginal cross-national differences. The mean willingness-to-pay index was modestly but significantly higher in the United Kingdom. Effect sizes indicate that these differences are generally small in magnitude, with the largest contrasts observed for trust-related variables. These differences underline the contrasting social-trust contexts within which consumers make decisions about organic food in each country.

**Table 3:** Comparison of key variables between the United Kingdom and Japan

Variable	UK M (SD)	JP M (SD)	t	p	d
Trust in government	2.68 (1.53)	3.10 (1.48)	− 7.27	< 0.001	−0.28
General trust	3.57 (1.61)	3.11 (1.55)	7.64	< 0.001	0.29
Risk appetite	4.85 (2.14)	4.64 (1.96)	2.72	0.007	0.10
Left–right ideology	4.85 (2.16)	4.99 (1.55)	− 1.83	0.067	−0.07
Willingness-to-pay index	0.46 (0.44)	0.40 (0.45)	3.48	0.001	0.13

Entries show means and standard deviations; t-tests report two-tailed significance levels; effect sizes are reported as Cohen’s d.

Next, we present the logistic regression analyses for the willingness-to-pay variables in Table 4, followed by the results for perceived quality and value alignment in Table 5. Our results for willingness to pay more for organic food are highly consistent across all food types.  Across both countries, trust in government is a consistently positive predictor of willingness to pay for organic food, with stronger and more uniform effects in Japan than in the United Kingdom. Generalised social trust also showed a positive association across both countries and all food types. The appetite for risk was also positively associated with a willingness to pay more across all food types in both countries. While the estimated effects are modest, typically corresponding to increases of only a few percentage points in the predicted probability of willingness to pay, they are consistent across food categories and national contexts, suggesting that the relationships are substantively meaningful even if small in magnitude.

**Table 4:** Willingness to pay more for organic food

	*Japan*					*UK*				
	Pay More for Dairy	Pay More for Beef	Pay More for Chicken	Pay More for Eggs	Pay More for Vegetables	Pay More for Dairy	Pay More for Beef	Pay More for Chicken	Pay More for Eggs	Pay More for Vegetables
General trust	0.137^***^	0.149^***^	0.131^***^	0.134^***^	0.128^***^	0.122^***^	0.090^**^	0.126^***^	0.114^***^	0.159^***^
	−0.038	−0.038	−0.038	−0.038	−0.037	−0.04	−0.04	−0.04	−0.039	−0.04
Trust government	0.152^***^	0.135^***^	0.115^***^	0.123^***^	0.094^**^	0.071^*^	0.119^***^	0.103^**^	0.105^**^	0.112^***^
	−0.039	−0.039	−0.039	−0.039	−0.039	−0.042	−0.042	−0.042	−0.042	−0.042
Take risks	0.088^***^	0.084^***^	0.075^**^	0.111^***^	0.114^***^	0.080^***^	0.127^***^	0.100^***^	0.081^***^	0.100^***^
	−0.03	−0.03	−0.03	−0.03	−0.029	−0.029	−0.03	−0.029	−0.029	−0.03
Left–right	−0.074^**^	−0.063^*^	−0.048	−0.038	−0.046	−0.048	−0.004	−0.022	−0.04	−0.070^**^
	−0.037	−0.037	−0.037	−0.037	−0.037	−0.031	−0.031	−0.031	−0.031	−0.031
Age	0.016^***^	0.011^***^	0.013^***^	0.020^***^	0.017^***^	−0.002	−0.009^**^	−0.004	−0.001	−0.005
	−0.003	−0.004	−0.003	−0.004	−0.003	−0.004	−0.004	−0.004	−0.004	−0.004
University	0.124	0.063	0.026	0.033	0.083	0.366^***^	0.394^***^	0.404^***^	0.455^***^	0.402^***^
	−0.113	−0.114	−0.112	−0.113	−0.111	−0.123	−0.124	−0.123	−0.123	−0.124
Women	0.126	0.051	0.087	0.147	0.077	0.324^***^	0.197	0.269^**^	0.424^***^	0.405^***^
	−0.114	−0.115	−0.114	−0.114	−0.113	−0.119	−0.121	−0.12	−0.12	−0.121
Constant	−2.335^***^	−2.173^***^	−2.086^***^	−2.608^***^	−2.206^***^	−1.179^***^	−1.421^***^	−1.372^***^	−1.286^***^	−1.261^***^
	−0.311	−0.313	−0.308	−0.314	−0.309	−0.301	−0.307	−0.303	−0.301	−0.304
Observations	1,478	1,475	1,475	1,469	1,458	1,263	1,263	1,263	1,263	1,263
Log Likelihood	−946.884	−934.96	−951.093	−946.135	−958.544	−843.366	−821.4	−839.757	−842.632	−828.916
Akaike Inf. Crit	1,909.77	1,885.92	1,918.19	1,908.27	1,933.09	1,702.73	1,658.80	1,695.51	1,701.26	1,673.83

^*^p < 0.1; ^**^p < 0.05; ^***^p < 0.01

**Table 5:** Quality and values

	*Dependent variable:*			
	*Japan*		*UK*	
	Organic quality	Organic values	Organic quality	Organic values
General trust	0.019	0.057^***^	0.004	0.088^***^
	(0.020)	(0.020)	(0.027)	(0.028)
Trust government	0.048^**^	0.057^***^	0.073^**^	0.052^*^
	(0.021)	(0.021)	(0.028)	(0.029)
Take risks	0.062^***^	0.052^***^	0.080^***^	0.048^**^
	(0.016)	(0.015)	(0.020)	(0.020)
Left–right	0.004	−0.032^*^	−0.014	−0.094^***^
	(0.020)	(0.019)	(0.021)	(0.021)
Age	−0.004^**^	0.002	−0.010^***^	−0.004
	(0.002)	(0.002)	(0.002)	(0.003)
University	−0.019	0.046	−0.148^*^	0.368^***^
	(0.061)	(0.060)	(0.085)	(0.087)
Women	0.292^***^	0.230^***^	0.316^***^	0.425^***^
	(0.061)	(0.060)	(0.081)	(0.084)
Constant	3.587^***^	3.267^***^	3.681^***^	3.998^***^
	(0.162)	(0.159)	(0.204)	(0.210)
Observations	1,471	1,475	1,263	1,263
R^2^	0.030	0.034	0.051	0.085
Adjusted R^2^	0.025	0.030	0.045	0.080
Residual Std. Error	1.131 (df = 1463)	1.115 (df = 1467)	1.405 (df = 1255)	1.447 (df = 1255)
F Statistic	6.456^***^ (df = 7; 1463)	7.487^***^ (df = 7; 1467)	9.583^***^ (df = 7; 1255)	16.677^***^ (df = 7; 1255)

^*^p < 0.1; ^**^p < 0.05; ^***^p < 0.01

Political opinion, measured by ideological (left–right) self-placement, shows only limited association with willingness to pay in both countries. Age is positively associated with willingness to pay more in Japan, but shows no significant association in the United Kingdom. University education is not associated with willingness to pay more for organic food in Japan, whereas in the United Kingdom the association is positive and statistically significant. In Japan, we estimate no systematic difference between men and women, while in the United Kingdom women are generally more willing to pay more for organic food than men, with the exception of organic beef. Taken together, these results suggest that institutional trust and individual risk orientation play consistent yet moderate roles in shaping consumer preferences, complementing rather than displacing the influence of socioeconomic characteristics.

These findings indicate that consumer responses differ between the two countries, highlighting the need to consider contextual factors. Whereas much prior research on organic consumption has emphasised attitudinal factors or the role of subjective norms, our findings highlight the centrality of institutional trust and individual risk appetite. For example, Nguyen and Nguyen (2025) demonstrate how behavioural reasoning and social influence explain the attitude-intention gap in Vietnam. Our results complement this line of work by showing that, across two contrasting national contexts, trust in government and willingness to take risks play consistent and meaningful roles in shaping consumers’ willingness to pay a premium for organic food.

Turning to perceptions of the consistent quality of organic food and the alignment of values of organic production methods and respondents’ personal values, we observe somewhat different patterns. Trust in government is positively associated with both outcomes, although the association is only marginally significant (*p* < 0.10) for value alignment among UK respondents. In both countries, generalised social trust is not significantly associated with perceptions of consistent quality; however, it is positively and significantly associated with beliefs that organic food reflects respondents’ personal values. Risk appetite shows a positive and statistically significant association with both perceived quality and value alignment in both questions.

## Discussion

Our findings provide broad support for the trust-risk framework. All three hypotheses in the first block (H1a–c) are supported: generalised social trust, trust in government, and risk appetite are each positively associated with a higher willingness to pay a premium for organic food in both the United Kingdom and Japan, with the effect of institutional trust particularly pronounced in Japan. While these relationships are modest in magnitude, their consistency across products and national contexts underscores the relevance of trust and risk orientations for understanding organic food preferences.

Moving to perceived product quality, only institutional trust (H2b) and risk appetite (H2c) retain statistical significance; generalised social trust (H2a) does not significantly influence assessments of quality in either country. Finally, when we treat organic purchasing as an expression of values, the pattern reverts: all three mechanisms are significant (H3a–c), although the government-trust coefficient is again stronger among Japanese respondents. In short, institutional trust is the most consistent driver across outcomes, while generalised trust matters chiefly for value alignment, and risk appetite operates steadily throughout.

We find clear empirical support for the idea that trust is a critical driver of organic food preferences, which is corroborated by recent studies (e.g. Macready et al 2025; Rashid and Lone 2024; Park-Poaps and Han 2025; Yuan et al. 2024). Specifically, trust in government emerges as a consistent and substantively important predictor of willingness to pay, belief in organic quality, and value alignment in both the UK and Japan. This supports findings by Murphy et al. (2022), Ayyub et al. (2021), and Hu et al. (2024), who identify trust in regulatory institutions as a key determinant of consumer confidence in organic labels. Generalised social trust, while also influential, shows more variation between countries. In the UK, where the organic market is relatively mature and institutional trust is more diffuse, generalised trust significantly predicts all outcomes, suggesting that interpersonal trust networks and social norms help embed organic food into consumer behaviour (Vega-Zamora et al. 2020; Sønderskov 2009). In Japan, where organic food is less established, generalised trust plays a secondary role, and institutional signals dominate (Yang et al. 2022; Tandon et al. 2021). Cross-national comparison reveals consistent differences in the magnitude and direction of trust effects. In Japan, trust in government is more consistently significant across all outcomes, whereas in the UK, both trust variables matter. This aligns with the literature on trust architectures in different cultural contexts (Sønderskov & Daugbjerg 2011; Yang et al. 2022).

These findings highlight the cultural specificity of trust effects. In high-context cultures like Japan, institutional authority often plays a more prominent role in legitimating consumer behaviour. In the UK, the decentralised nature of organic certification and a more individualistic culture may amplify the relevance of general social trust. The control variables behaved largely as expected. Women and respondents with higher levels of education were more favourable toward organic food, consistent with previous research (Gumber and Rana 2021; Kowalska et al. 2021; Kushwah et al. 2019). Age effects varied by country, while political orientation was more influential in the UK, where left-leaning respondents expressed stronger organic preferences, consistent with Tiganis et al. (2023).

Our overall research design is subject to several limitations. Most importantly, we do not present experimental evidence, meaning that the relationships identified in this study should be interpreted as associational rather than causal. We also do not include a direct measure of respondents’financial circumstances. Income variables in survey research are often affected by item non-response or over-reporting (see Hariri and Lassen 2017; Neri and Porreca 2024), and these issues cannot be fully addressed within the present survey design. For this reason, we exclude income from the analysis and acknowledge that our findings should be interpreted in light of this omission. We also note that, however, that our willingness-to-pay measure captures respondents’ stated willingness rather than their objective ability to pay. A further limitation concerns case selection. The United Kingdom and Japan are both highly-developed economies, and our findings may not generalise to countries at different stages of economic or institutional development.

We also note that several other limitations should be considered when interpreting our results. First, key constructs such as generalised trust, trust in government and risk appetite were each measured with single-item indicators. While this approach is common in large-scale survey research, it necessarily limits the precision with which these concepts can be captured. Future work could employ multi-item scales to assess whether the observed associations remain consistent when measurement reliability is increased.

Second, our models focus on trust and risk as predictors of organic food preferences and do not include some well-established correlates of organic consumption, such as environmental concern, health motivations, or income. These variables are likely to explain additional variance in willingness to pay, and their inclusion would offer a fuller account of the mechanisms linking social trust and consumer behaviour. Nevertheless, the consistent direction and broadly similar magnitude of effects across two distinct national contexts lends confidence to the robustness of our main findings.

## Conclusion

This study provides clear empirical evidence that trust (both general and institutional) is an important factor in shaping consumer preferences for organic food. While trust in government emerges as the most consistent correlate across both countries, generalised trust appeared more influential in the UK, highlighting the role of contextual trust architectures. Risk appetite is also positively associated with all outcomes, underscoring the behavioural dimension of organic purchasing. Although the effect sizes are modest, their consistency across products and national contexts suggests that institutional confidence and individual risk orientation provide stable foundations for understanding sustainable consumption.

Our findings make three main contributions. Theoretically, we clarify how trust functions in different institutional environments, showing that even when organic food is framed as an ethical or health-related choice, its uptake is closely linked to credible intermediaries. In Japan, where certification is centralised, institutional signals matter more; in the UK, where certification is fragmented, generalised social trust plays a bigger role. Methodologically, we leverage harmonised, large-scale survey data to directly compare two distinct food cultures. Empirically, we show that these psychological drivers remain relevant even when controlling for socio-demographics and political ideology.

Practically, these findings suggest that strategies to expand organic food markets should be tailored to national trust profiles. In contexts like Japan, strengthening the visibility and transparency of state-led certification may encourage uptake. In the UK, working through social networks, trusted NGOs, and third-party certification schemes may prove more effective.

There are limitations. Our data are cross-sectional and self-reported, and the analysis does not include income or attitudinal measures such as environmental concern. Even so, the consistent direction of effects across two contrasting institutional contexts lends support to the stability of our conclusions. Future work could examine how trust evolves in response to food scares, certification controversies, or institutional reform. Experimental or longitudinal designs would help untangle causal dynamics, while extending this research to low- and middle-income countries would provide a stronger test of its global applicability.

In doing so, this line of research can contribute to UN Sustainable Development Goal 12 (Responsible Consumption and Production) by clarifying how trust and risk perceptions shape everyday choices about food and sustainability. Trust may be intangible, but as our results show, its effects are very real at the grocery checkout.

## Data Availability

Full replication data and code are available from the Harvard Dataverse, at: [10.7910/DVN/Q8KOW8].
